# Inter-Organelle Crosstalk in Oxidative Distress: A Unified TRPM2-NOX2 Mediated Vicious Cycle Involving Ca^2+^, Zn^2+^, and ROS Amplification

**DOI:** 10.3390/antiox14070776

**Published:** 2025-06-24

**Authors:** Esra Elhashmi Shitaw, Maali AlAhmad, Asipu Sivaprasadarao

**Affiliations:** 1 School of Biomedical Sciences, Faculty of Biological Sciences, University of Leeds, Leeds LS2 9JT, UK; fy14ees@leeds.ac.uk; 2 Department of Biological Sciences, College of Science, Kuwait University, Alshadadiya, P.O. Box 5969, Safat 130602, Kuwait; maali.alahmad@ku.edu.kw

**Keywords:** oxidative stress, reactive oxygen species, TRPM2, NOX2, PARP, calcium, zinc, mitochondria, lysosomes, inter-organelle communication, noncommunicable diseases

## Abstract

Reactive oxygen species (ROS) are critical signalling molecules, but their overproduction leads to oxidative stress (OS), a common denominator in the pathogenesis of numerous non-communicable diseases (NCDs) and aging. General antioxidant therapies have largely been unsuccessful, highlighting the need for a deeper understanding of ROS amplification mechanisms to develop targeted interventions. This review proposes a unified, self-amplifying “vicious cycle” of inter-organelle crosstalk that drives pathological ROS elevation and cellular damage. We outline a pathway initiated by extracellular stressors that co-activate plasma membrane TRPM2 channels and NADPH oxidase-2. This synergy elevates cytoplasmic Ca^2+^, leading to lysosomal dysfunction and permeabilization, which in turn releases sequestered Zn^2+^. Mitochondrial uptake of this labile Zn^2+^ impairs electron transport chain function, particularly at Complex III, resulting in mitochondrial fragmentation, loss of membrane potential and a burst of mitochondrial ROS (mtROS). These mtROS diffuse to the nucleus, activating PARP-1 and generating ADPR, which further stimulates TRPM2, thereby perpetuating the cycle. This “circular domino effect” integrates signals generated across the plasma membrane (Ca^2+^), lysosomes (Zn^2+^), mitochondria (ROS) and nucleus (ADPR), leading to progressive organelle failure, cellular dysfunction, and ultimately cell death. Understanding and targeting specific nodes within this TRPM2-NOX2-Ca^2+^-Zn^2+^-mtROS-ADPR axis offers novel therapeutic avenues for NCDs by selectively disrupting pathological ROS amplification while preserving essential physiological redox signalling.

## 1. Introduction

Non-communicable diseases (NCDs), such as cardiovascular disease, diabetes, cancer, and neurodegenerative disorders, represent the foremost global health challenge of the 21st century. According to the World Health Organization, NCDs are responsible for a staggering 74% of all deaths worldwide, claiming 41 million lives annually (Ten threats to global health in 2019). This immense health burden places an unsustainable strain on healthcare systems and the global economy [1,2]. The impact is disproportionately severe in low- and middle-income countries, where over three-quarters of all NCD deaths occur, threatening to reverse decades of development progress [3]. A common thread weaving through the pathogenesis of many of these diverse conditions is chronic oxidative stress (OS)—a state where the production of reactive oxygen species (ROS) overwhelms the body’s antioxidant defences [4,5,6,7,8,9,10,11,12,13,14]. However, the failure of general antioxidant therapies has highlighted a critical gap in our understanding. It is not simply the presence of ROS, but the specific mechanisms that amplify them to pathological levels, that must be targeted [8]. This review synthesizes recent evidence to propose a novel, unified mechanism that drives this pathological amplification, offering a new perspective on the shared origins of NCDs and potential avenues for targeted intervention.

### 1.1. The Double-Edged Sword of ROS

ROS are oxygen-derived molecules with dual roles in biology. At low-to-moderate concentrations, they serve as essential signalling molecules in physiological processes, but at high concentrations, they become detrimental, contributing to pathology [4,5,6,7,8,9,10,11,12,13,14]. Cellular ROS levels can be elevated by exogenous factors (e.g., toxins, xenobiotics, radiation) and endogenous stressors (e.g., metabolic dysfunction, inflammation), potentially overwhelming the cell’s antioxidant capacity and inducing OS. Cellular OS is recognized as a common factor underlying the pathogenesis of numerous NCDs, including cardiovascular disorders [6,15,16,17], neurodegenerative diseases like Parkinson’s [18,19,20] and Alzheimer’s [21], diabetes mellitus [22,23], aging [6,15,16,17,24,25], and various cancers [26].

Current therapeutic strategies typically address NCDs individually. However, targeting the shared pathology of OS could offer a more comprehensive approach [6,8,27]. Despite this rationale, clinical trials employing general antioxidant therapies (e.g., vitamins C and E, glutathione precursors) have largely failed to demonstrate significant clinical benefits [5,6,8,12,28,29]. A primary reason for this failure is likely their non-specific scavenging action, which reduces detrimental ROS but also impairs essential physiological ROS signalling. Other reasons arise from the physicochemical properties of antioxidant supplements, which can prevent them from reaching the target site at the optimal time [30]. Additionally, these properties can paradoxically exacerbate the issue by transforming the supplements into prooxidants [29,30,31]. This highlights the critical need to identify and selectively target the specific mechanisms that amplify ROS to pathogenic concentrations, thereby preserving necessary basal ROS functions. This review delineates how ionic signals (Ca^2+^ and Zn^2+^) orchestrate inter-organelle crosstalk between the plasma membrane, lysosomes, mitochondria and the nucleus to exacerbate ROS production and inflict organelle and cellular damage. We will conclude by discussing the potential of targeting this newly elucidated signalling axis to develop broad-spectrum therapeutics capable of mitigating multiple OS-associated diseases and potentially improving the healthspan-to-lifespan ratio during aging.

### 1.2. Maintaining Redox Balance: Production vs. Defence

ROS encompass various reactive oxygen derivatives, including free radicals (e.g., superoxide, O_2_^•−^; hydroxyl radical, ^•^OH) and non-radicals (e.g., hydrogen peroxide, H_2_O_2_). While O_2_^•−^ and particularly H_2_O_2_ are key mediators of physiological redox signalling, the highly reactive ^•^OH is primarily implicated in oxidative damage [5,6,8,9,10,11,12,13,14,29]. Healthy cells meticulously maintain redox homeostasis—a dynamic balance between ROS production and elimination. ROS are physiologically generated at numerous subcellular sites, including mitochondria (primarily the electron transport chain), peroxisomes, the endoplasmic reticulum, and by plasma membrane-associated NADPH oxidases (NOX enzymes), contributing to processes like cell proliferation and differentiation, host defence, and metabolic adaptation. However, under stress conditions, some sites can significantly increase their ROS generation rate [6,8,9,10,11,12,13,14,29].

To counteract excessive ROS accumulation, cells possess sophisticated defence mechanisms [6,8,14,25,29,32,33]. Firstly, antioxidant enzymes rapidly metabolise ROS, including superoxide dismutases (SOD1-3, converting O_2_^•−^ to H_2_O_2_), catalase and glutathione peroxidases (neutralizing H_2_O_2_), and peroxiredoxins (Prx1-6) alongside the thioredoxin system (reducing peroxides). Secondly, non-enzymatic antioxidants like glutathione (GSH), vitamins C and E, coenzyme Q10, NADPH, and bilirubin directly neutralize ROS. Thirdly, organelle membranes (mitochondria, peroxisomes, endosomes, ER) act as physical barriers limiting the diffusion of highly reactive species like O_2_^•−^ and ^•^OH, restricting unwanted cytoplasmic oxidation. Ultimately, whether ROS exert physiological signalling effects (eustress) or cause detrimental damage (distress) depends critically on the specific ROS molecule, its concentration, subcellular location, and lifetime.

**Figure 1:**
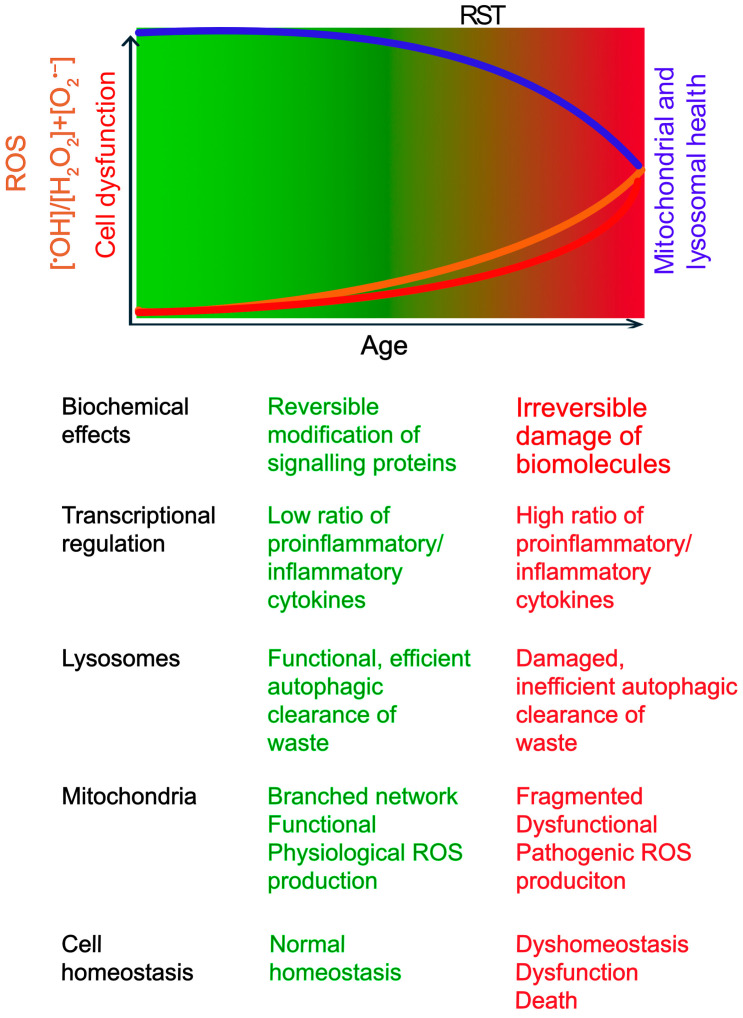
**The Concept of Redox Stress Signalling Threshold (RST) in Aging and Disease.** This schematic illustrates the concept of RST. The gradient represents the increase in oxidative stress with age. RST is a conceptual boundary marking the transition from oxidative eustress (green) to distress (red). As age progresses, ROS (Reactive Oxygen Species) levels (orange line) gradually rise, but any decline in mitochondrial and lysosomal functions (blue line) may not significantly impact cell function and viability (red line), until ROS production exceeds the RST. The age of onset varies based on genetic and environmental factors. Below the schematic, key biochemical and cellular changes associated with low (eustress) and high (distress) ROS levels are summarized.

From Eustress to Distress: Crossing the Redox Signalling Threshold ROS exhibit a dichotomous role, acting as both essential signals and potential mediators of damage. This duality is sometimes conceptualized by the “redox stress signalling threshold” (RST), distinguishing beneficial “oxidative eustress” from harmful “oxidative distress” [34] (Figure 1). For clarity, this review primarily uses the historically used term, “oxidative stress” to refer to oxidative distress.

Below the RST, physiological ROS signalling supports cellular homeostasis and adaptation [14,34]. This involves regulating enzyme activity and gene expression through reversible oxidation of critical cysteine thiols (-SH) in regulatory proteins, primarily mediated by species like H_2_O_2_. Key adaptive pathways include those governed by transcription factors Nrf2 (nuclear factor erythroid 2-related factor 2), which upregulates antioxidant defences, and AP-1 (activator protein 1), involved in proliferation and differentiation. Physiological ROS levels can also activate DNA repair enzymes to sustain genomic stability [14,34].

However, with age, susceptibility to chronic diseases increases. This is associated with an increase in ROS levels beyond the RST towards oxidative distress, characterized by an increased ratio of highly reactive species like ^•^OH relative to signalling molecules like H_2_O_2_ [14,34]. Unlike reversible modifications in eustress, ^•^OH causes irreversible damage, including DNA strand breaks, protein oxidation leading to misfolding/aggregation, and lipid peroxidation compromising membrane integrity [9,12,14]. Consequently, gene expression patterns shift, often favouring pro-inflammatory pathways (e.g., via NF-kB activation) while potentially suppressing adaptive responses governed by factors like Nrf2 [9,14,34]. This maladaptive shift, by overwhelming repair mechanisms and chronically activating damaging pathways, accelerates cellular dysfunction, promotes chronic inflammation, contributes to aging, and underlies many age-related diseases [9,14,34]. Notably, in conditions with genetic predispositions or specific environmental triggers (e.g., Type 1 diabetes, Parkinson’s disease), these detrimental changes can manifest earlier in life [18,23].

### 1.3. Mitochondrial and Lysosomal Contributions to Oxidative Distress

Accumulating evidence indicates that mitochondria and lysosomes play crucial roles in ROS signalling in both health and disease [23,35,36,37,38,39,40,41,42,43,44,45]. The quality, quantity, and function of mitochondria decline with aging and chronic diseases as ROS levels increase [24,35,38,44,46]. In healthy cells, mitochondrial health is maintained through a dynamic balance of fission (division) and fusion (merging) for quality control [24,38,44]. Fission segregates damaged components, while fusion mixes contents to restore function. Dysfunctional mitochondrial fragments are typically removed by lysosomes via selective autophagy (mitophagy) [24,38,44]. The lost mitochondrial density and quality is restored by the biogenesis of new mitochondria, regulated by PGC1-α (Peroxisome proliferator-activated receptor-gamma coactivator 1-alpha) transcription of genes involved in mitochondrial quality control [24,38,47]. PGC-1α interacts with and coactivates multiple DNA-binding transcription factors (e.g., NRF-1/2, Estrogen-Related Receptor alpha (ERRα), Mitochondrial Transcription Factor A (Tfam)) to control virtually all aspects of biogenesis, dynamics, and maintenance of mitochondrial protein and DNA levels [48].

Similarly, lysosomes undergo fusion and fission. They fuse with other organelles to acquire essential components and acidity required for their function [49,50]. Dysfunctional lysosomes are removed by selective autophagy (lysophagy) [51,52]. Lost lysosomes are replaced through biosynthesis, where the Ca^2+^-dependent transcription factor TFEB (Transcription Factor EB) plays a crucial role [53,54,55]. Thus, dynamic regulation of both mitochondrial networks and lysosomes maintains their density, shape, size, composition, and function. This dynamic regulation is compromised during aging and chronic diseases, leading to a decline in the structural and functional integrity of both organelles [24,35,37,38,44,49,50,56,57,58].

Excessive mitochondrial fragmentation, bioenergetic failure, and increased ROS production are well-established hallmarks of aging and numerous diseases. Similarly, a decline in lysosomal numbers and function are emerging features of many diseases, especially neurodegenerative diseases [36,37,42,45,46,53,56,59,60,61]. Notably, the two organelles support each other in quality control [62,63]. Mitochondria supply ATP to support lysosomal function, while lysosomes clear dysfunctional mitochondrial debris, supporting efficient bioenergetic function of mitochondria. This cooperation is gradually lost during aging and various diseases, resulting in both organelles contributing to damaging ROS production rather than supporting each other’s function [55,62,63,64,65,66]. Paradoxically, the excess ROS they generate results in their own destruction.

Compelling evidence links coupled mitochondrial and lysosomal dysfunction to ROS imbalance and chronic disease pathogenesis, particularly neurodegenerative disorders like Parkinson’s disease (PD) and Alzheimer’s disease (AD). Familial PD forms are associated with mutations in genes critical for mitochondrial dynamics and quality control (e.g., *PARK2* encoding Parkin, *PINK1*) and lysosomal function or autophagy (e.g., *GBA1* encoding β-glucocerebrosidase [36,67,68], *PARK9* encoding ATP13A2) [57,69]. Mutations affecting mitochondrial integrity impact lysosomes, and conversely, lysosomal protein mutations affect mitochondria, reflecting their functional relationship. Pathological oxidative stress impact is evident in post-mortem PD brains, showing accumulated ROS-damaged biomolecules and autophagic vacuoles [70]. Excessive mitochondrial ROS production and defective mitophagy also characterise idiopathic (sporadic) PD [71]. A recent seminal study has reported mitochondrial plaques (spatially associated with Aβ plaques) within or outside of lysosomes in AD mouse models and human brains [64]. These studies highlight the importance of investigating the interplay between mitochondria and lysosomes, rather than studying each separately.

A critical, incompletely answered question is the precise mechanism triggering the switch from physiological ROS production to sustained, pathological ROS amplification across the redox signalling threshold in diverse chronic stress conditions. An associated question is how this is linked to the interplay between mitochondria and lysosomes. Addressing these questions is paramount for developing novel therapeutic strategies that selectively curb excessive ROS production while preserving essential basal redox signalling. The following sections review key cellular ROS sources and proposed mechanisms driving pathological amplification.

### 1.4. Cellular Sources of Reactive Oxygen Species

Cells contain numerous redox-active proteins capable of transferring electrons, often from NADH and NADPH, to molecular oxygen (O_2_), initially generating the superoxide radical (O_2_^•−^). This O_2_^•−^ can then be converted enzymatically (e.g., by SODs) or non-enzymatically into other ROS, including signalling H_2_O_2_ or damaging ^•^OH. Many ROS-generating proteins are membrane-associated and utilize redox-active metal ions (Fe^2+^/Fe^3+^ or Cu^+^/Cu^2+^) or organic cofactors (FMN, FAD, heme, pterins). Among the most significant contributors, particularly in pathology, are the NADPH oxidase (NOX) enzyme family and the mitochondrial electron transport chain (ETC) respiratory complexes. Lysosomes also contribute significantly to ROS production, albeit indirectly.

#### 1.4.1. NADPH Oxidases (NOX Enzymes)

**Figure 2:**
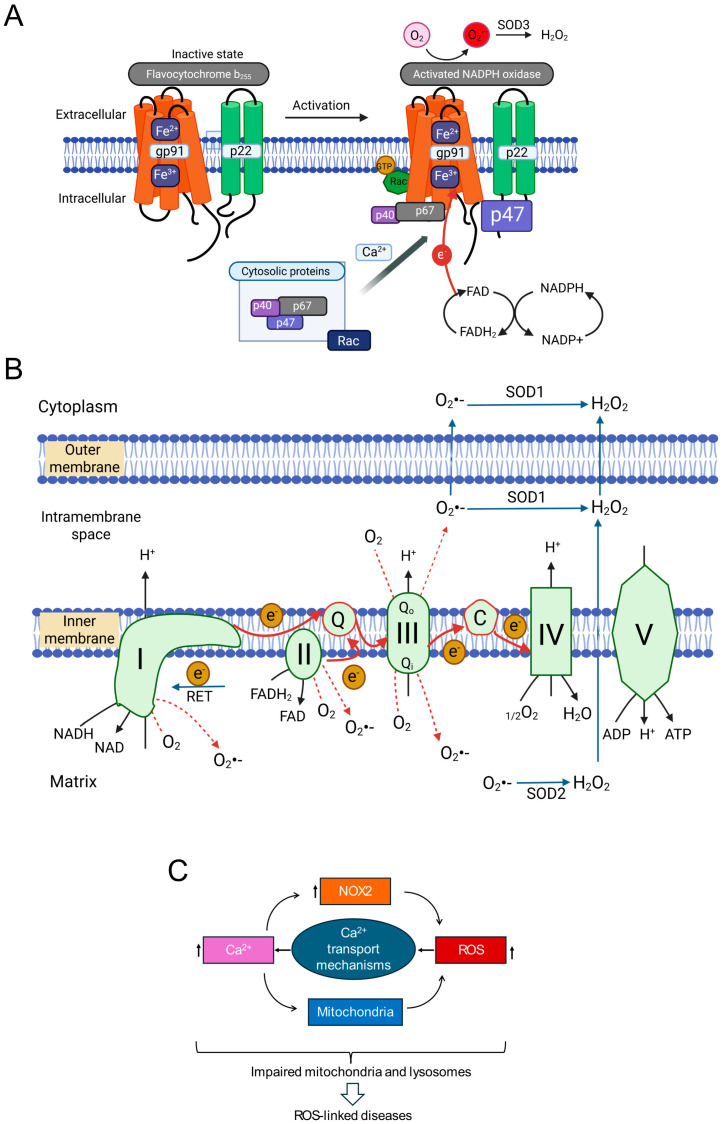
**Key Components Involved in ROS-induced ROS production (RIRP) and Ionic Signalling** (**A**) NADPH oxidase 2 (NOX2) activation mechanism. Cytoplasmic Ca^2+^ (among other stimuli) promotes translocation of regulatory subunits (p47^phox^, p67^phox^, Rac) to join the membrane-bound catalytic core (gp91^phox^/p22^phox^). The assembled complex transfers electrons from NADPH via FAD/heme to extracellular O_2_, producing O_2_^•−^, which is converted to H_2_O_2_ by SOD1 (superoxide dismutase-1) and can enter the cell. (**B**) Mitochondrial ROS production. Schematic of the electron transport chain showing electron flow (red arrows) and sites of electron leak generating O_2_^•−^. Forward Electron Transport (FET) occurs during normal respiration and stress. Reverse Electron Transport (RET) can occur under high ΔΨmt (mitochondrial membrane potential). O_2_^•−^ generated in the matrix (Complex I leak) is converted to H_2_O_2_ by SOD2. O_2_^•−^ generated in the intermembrane space (Complex III leak) can exit via porins or be converted to H_2_O_2_ (by SOD1 or SOD2) before diffusing into the cytoplasm via aquaporins. (**C**) Conceptual framework showing interplay between Ca^2+^, NOX2, and mitochondria in ROS-induced ROS production. Increased cytoplasmic Ca^2+^ stimulates ROS from both NOX2 and mitochondria. ROS, in turn, can enhance Ca^2+^ signals, forming potential positive feedback loops contributing to pathological ROS amplification.

The human NOX family comprises NOX1-5 and the dual oxidases DUOX1/2 [72,73,74]. These transmembrane proteins primarily function in regulated ROS generation. All NOX enzymes share a core structure with a membrane-embedded domain housing heme groups and a cytoplasmic dehydrogenase domain containing NADPH and FAD binding sites. This facilitates electron transfer from NADPH via FAD to heme groups, which then transfer electrons across the membrane to O_2_, producing O_2_^•−^ (Figure 2A) [72,74,75]. DUOX proteins possess additional domains.

From a pathophysiological perspective, NOX2 (gp91phox) is particularly important. Unlike some isoforms, NOX2 activity requires multi-subunit complex assembly. In resting cells, the catalytic subunit (gp91^phox^) resides in the membrane, often with p22^phox^. Regulatory subunits (p40^phox^, p47^phox^, p67^phox^, and Rac GTPase) are cytoplasmic. Upon stimulation (e.g., increased intracellular Ca^2+^), these cytosolic subunits translocate and assemble with the core, forming the active enzyme. Activated NOX2 typically releases O_2_^•−^ extracellularly. Extracellular SOD3 converts this to H_2_O_2_, which can enter the cytoplasm via aquaporins or diffusion [72,73,74,75]. While crucial for phagocytic host defence [76], accumulating evidence implicates NOX2-derived ROS in NCD pathogenesis, including inflammation, metabolic disorders, cardiovascular diseases, and neurodegeneration [72,73,74,77,78].

#### 1.4.2. Mitochondrial Electron Transport Chain

Mitochondria are metabolic hubs and major producers of ROS. Most mitochondrial ROS (mtROS) originate from electrons leaking prematurely from ETC complexes (I, II, III, IV) in the inner mitochondrial membrane (IMM) (Figure 2B). During oxidative phosphorylation, electrons from NADH and FADH_2_ pass along the ETC to O_2_, reducing it to H_2_O at Complex IV. This electron flow drives proton pumping, generating the mitochondrial membrane potential (ΔΨmt) for ATP synthesis [4,79,80].

Electron escape primarily occurs from intermediates within Complex I (NADH:ubiquinone oxidoreductase) and Complex III (ubiquinol:cytochrome c oxidoreductase), reacting directly with O_2_ to produce O_2_^•−^ (forward electron transport, FET leakage). Under conditions like ischemia-reperfusion (substrate accumulation, high mitochondrial membrane potential), electrons can flow backward from Complex II through Complex I (reverse electron transport, RET), becoming a major source of O_2_^•−^ from Complex I [4,81]. Determining the relative contributions of FET (Complexes I/III) versus RET (Complex I) to signalling is challenging due to the functional and physical linkage of these complexes within the IMM. Knockout studies showed that genetic deletion of one complex can impact ROS production from the other [82], while biochemical studies reported that complex III supports the assembly of individual complexes into a super-complex [83].

Although historically the focus has been on complex I, recent studies highlight the role of Complex III in ROS generation during chronic stress [84,85,86,87,88,89]. Ubiquinone facilitates electron transfer from Complexes I and II to Complex III by transitioning between its oxidized form (ubiquinone, Q) and its reduced form (ubiquinol, QH_2_) via a semiquinone intermediate (Q^•−^). Chronic stress by reducing mitochondrial membrane potential (ΔΨmt) can alter the redox state of the ubiquinone pool, increasing the lifespan of reactive Q^•−^, leading to increased electron leak and O_2_^•−^ production. Reduced ΔΨmt (depolarization) may favour FET-based ROS production from Complex III over RET from Complex I, suggesting that mechanisms of ROS production may be different between chronic and acute stress conditions. Although the recent pharmacological tools (e.g., S1QELs for Complex I, S3QELs for Complex III) have provided valuable insights into the roles of complexes [10,90], complex feedback mechanisms between the complexes might complicate the interpretation of the findings [82,83].

A key difference between the two complexes is the topology of ROS release. Complex I releases O_2_^•−^ to the matrix side, while Complex III has two sites (Qo and Qi) that release O_2_^•−^ to the intermembrane space (IMS) and matrix, respectively. Most O_2_^•−^ from Complex III is released into the IMS, where it can be converted to H_2_O_2_ by IMS-localized SOD1 and diffuse into the cytoplasm [91,92]. Some O_2_^•−^ may exit into the cytoplasm via the outer membrane VDAC (voltage dependent anion channel) [91]. This suggests Complex III might more readily release damaging ROS into the cytoplasm under chronic stress. Thus, Complexes I and III are primary ETC sites implicated in enhanced ROS production during stress, with context-dependent mechanisms and contributions.

#### 1.4.3. Lysosomes: Indirect Contributors to ROS Generation

As mentioned, dysfunctional lysosomes contribute significantly to cellular ROS burden indirectly and disease aetiology. By failing to clear autophagosomes containing damaged mitochondria, protein aggregates (which can trigger ROS or sequester antioxidants), or damaged lysosomes themselves, they allow OS sources to accumulate [36,37,46,49,59,61,66,67]. Clinical evidence supports this: protein aggregate accumulation (Aβ, α-synuclein, mutant Huntingtin) and autophagosome buildup in neurodegenerative diseases are linked to impaired lysosomal-autophagic clearance and increased OS [36,37,59,61,67,93].

Interestingly, lysosomes sense and respond to acute ROS increases. ROS can trigger Ca^2+^ release from lysosomal stores via the MCOLN1 (TRPML1) channel [94], which acts as a ROS sensor. This lysosomal Ca^2+^ release can activate TFEB, a master regulator of lysosomal biogenesis and autophagy, potentially representing a protective feedback mechanism [46,67]. However, under chronic, overwhelming OS, this adaptive response may become insufficient.

Lysosomes can also be minor direct ROS sources during enzymatic degradation. More critically, severe or chronic OS can destabilize lysosomal membranes, causing lysosomal membrane permeabilization (LMP) or rupture resulting in the release of acidic hydrolases (e.g., cathepsins) and stored metal ions (Fe^2+^/Fe^3+^, Zn^2+^) into the cytoplasm. Released cathepsins can cleave proteins, activate apoptosis, and trigger inflammasomes, promoting inflammatory responses that further increase ROS. Released ferrous iron (Fe^2+^) is particularly dangerous, catalysing the Fenton reaction with H_2_O_2_ to generate highly damaging ^•^OH [12,29]. These findings highlight that maintaining both mitochondrial and lysosomal integrity is crucial for preventing runaway OS and disease progression.

### 1.5. Amplifying the Damage: ROS-Induced ROS Production (RIRP)

It is increasingly evident that the simultaneous or sequential activation of multiple ROS sources, coupled with positive feedback, is often necessary to elevate ROS levels to pathogenic concentrations. A key concept is “ROS-induced ROS production” (RIRP), where an initial oxidative insult triggers cellular responses that lead to further ROS generation, creating a self-perpetuating vicious cycle. This feedback loop is believed to significantly contribute to aging and many chronic diseases [95].

Given the multiple cellular ROS sources, numerous RIRP pathways involving crosstalk between organelles and enzymes are possible. However, emerging evidence highlights a particularly important self-amplifying loop involving the interplay between NOX enzymes (especially NOX2) and mitochondrial ROS production [88,95,96]. In this loop, NOX-generated ROS stimulates mitochondrial ROS (mtROS) and vice versa. Supporting the causal role of NOX-mitochondria interplay in (OS-linked diseases, therapeutic strategies that interrupt this cycle (e.g., specific NOX inhibitors, mitochondria-targeted antioxidants like MitoQ) [6,8,28,29,97] show robust preclinical promise. However, clinical translation faces hurdles: achieving selectivity for pathological sources, ensuring bioavailability, and crucially, avoiding the suppression of essential physiological ROS signalling. Similarly, trials using general antioxidants have largely failed, likely due to their inability to selectively target pathological ROS overproduction and potential interference with essential signalling [12,28,29].

These observations underscore the clinical need for a deeper mechanistic understanding of RIRP. Specifically, how does NOX-derived ROS signal to mitochondria to stimulate mtROS, and how does mtROS feedback to sustain NOX activity or activate other sources? Elucidating these pathways is crucial for designing more effective, selective therapies that break the pathological ROS amplification cycle without disrupting normal redox homeostasis.

## 2. Ionic Messengers in RIRP: The Roles of Ca^2+^ and Zn^2+^

Intricate crosstalk between calcium (Ca^2+^) signalling and ROS production is well-established [98,99]. Ca^2+^ and ROS often form a positive feedback loop: elevated intracellular Ca^2+^ can stimulate ROS production (from mitochondria, NOX enzymes), while ROS can modulate Ca^2+^ channels and pumps, further increasing intracellular Ca^2+^ [99]. Given that Ca^2+^ activates/modulates both NOX2 and mitochondrial metabolism (influencing mtROS), integrating Ca^2+^-ROS crosstalk into the RIRP framework is logical. This suggests a potential three-way positive feedback loop involving NOX2-derived ROS, mtROS, and intracellular Ca^2+^ signals (Figure 2C), potentially driving a self-reinforcing cycle in pathology.

Several Ca^2+^ channel types mediate ROS effects on Ca^2+^ influx or release, including voltage-gated channels, ligand-gated channels (NMDA receptors), store-operated calcium entry (SOCE) channels, and Transient Receptor Potential (TRP) channels [98,99]. Among TRP channels, TRPM2 (Transient Receptor Potential Melastatin 2) emerges as particularly important due to its direct sensitivity to OS-generated molecules and its established role in promoting OS-related damage in numerous cardiovascular, neurodegenerative, and metabolic disease models [100,101,102,103,104,105,106,107].

### 2.1. The TRPM2 Channel: A Key Ca^2+^ Conduit in Oxidative Stress

TRPM2 is a non-selective cation channel (permeable to Na^+^, K^+^, and notably, Ca^2+^) belonging to the Melastatin TRP subfamily. Functional channels are tetramers of identical subunits, each with six transmembrane segments (S1–S6) and large intracellular N- and C-termini. Uniquely, TRPM2 gating is synergistically activated by adenosine diphosphate ribose (ADPR) binding to the C-terminal NUDT9-H domain and Ca^2+^ binding to S2-S3 loop [103,104,108,109,110]. ADPR production significantly increases during OS. Oxidants like H_2_O_2_ cause DNA damage, activating poly(ADP-ribose) polymerase (PARP) enzymes. PARP uses NAD^+^ to generate poly(ADP-ribose) polymers; subsequent hydrolysis releases free ADPR. Thus, OS indirectly increases intracellular ADPR, which, with permissive intracellular Ca^2+^ concentrations, triggers TRPM2 opening and extracellular Ca^2+^ influx [103,104,109].

TRPM2 channels are expressed widely (neurons, cardiomyocytes, pancreatic β-cells, kidney, liver, endothelial cells, immune cells) and implicated in diverse processes (temperature sensation, immune activation, insulin secretion, apoptosis) [100,103,104,105,111]. Paradoxically, TRPM2 activation, requiring ROS-dependent ADPR generation, often leads to downstream events that further exacerbate ROS production in most cell types examined [88,112,113]. This ability to amplify OS appears central to its role in mediating OS-induced cellular dysfunction and apoptosis in various NCDs, including neurodegenerative, cardiovascular and metabolic diseases. Additionally, TRPM2 channels upregulated in cancer and their activation is linked to cancer cell proliferation and metastasis [114,115,116].

### 2.2. Zinc Dyshomeostasis and Mitochondrial ROS

Besides Ca^2+^, considerable evidence implicates zinc (Zn^2+^) dyshomeostasis in mitochondrial dysfunction and ROS production in OS-linked pathologies [117,118,119,120,121,122,123,124]. Zn^2+^ is an essential micronutrient, acting as a cofactor or structural component for numerous proteins (potentially > 2000), critical for enzyme catalysis, protein structure, gene transcription, and signalling. Most intracellular Zn^2+^ is tightly bound (e.g., to metallothioneins, high-capacity buffers) or sequestered within organelles (ER, Golgi, synaptic vesicles, lysosomes) [117,122,123,125]. Consequently, free cytoplasmic Zn^2+^ concentration is maintained at very low levels (picomolar to low nanomolar) physiologically [117,119,120,122,123]. This tight control involves coordinated action of Zn^2+^ transporters: ZIP family (SLC39A) generally facilitates cytoplasmic influx, while ZnT family (SLC30A) promotes cytoplasmic efflux [117,125].

Although essential, disruptions leading to either deficiency or excess labile Zn^2+^ in specific compartments are strongly linked to pathology. Notably, elevated intracellular Zn^2+^ is frequently associated with increased mtROS and subsequent cell damage/death in models of neurodegeneration, stroke, and other OS-related conditions [117,123,125,126,127]. The precise mechanisms by which excess Zn^2+^ leads to mtROS have been of considerable interest.

## 3. Integrating the Pieces: Towards a Unified Mechanism

Previous sections established that common hallmarks of stress responses in aging and related diseases include elevated OS (potentially RIRP-driven), mitochondrial damage (fragmentation, decline) [6,35,38,39,58,128,129], lysosomal impairment (defective autophagy, leakage) [37,42,46,49,53,61,65,67,130], and disrupted ionic signalling (Ca^2+^, Zn^2+^) [98,99,131,132] [117,119,121,123]. Literature provides evidence for pairwise connections: lysosome-mitochondria interplay [55,62,63,66]; NOX-mitochondria crosstalk in RIRP [88,96,133]; Ca^2+^-ROS positive feedback [99]. However, a key unresolved question is whether these disparate events are interconnected through a common underlying pathway. Could a single integrated mechanism explain how an initial stressor triggers this detrimental cascade across multiple organelles? Herein, we synthesize recent research to propose a unified mechanism integrating various signalling molecules with inter-organelle crosstalk to promote ROS amplification to cytotoxic levels.

### 3.1. A Unified Vicious Cycle: Inter-Organelle Crosstalk Drives Pathological ROS Amplification

**Figure 3:**
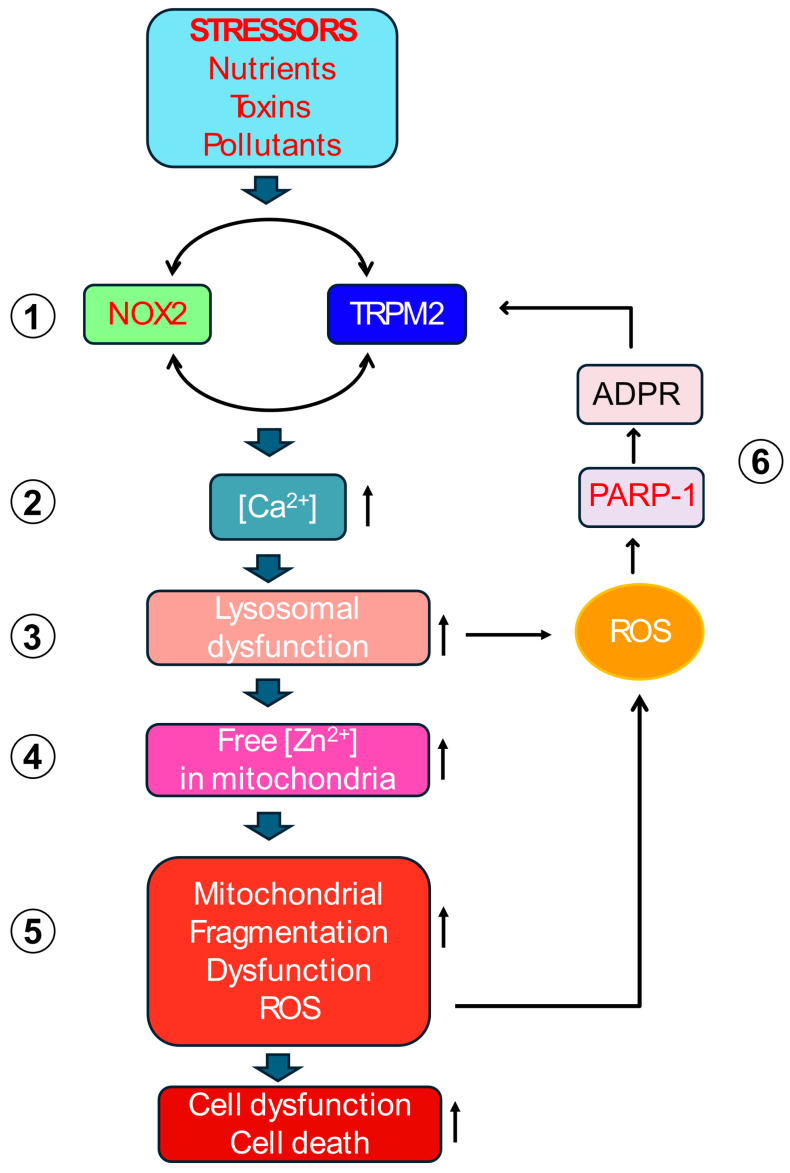
**The Proposed Self-Amplifying Cytotoxic Signalling Circuit**. Detailed schematic illustrating the proposed inter-organelle signalling cycle. (1) Extracellular stress activates plasma membrane TRPM2 and NOX2. (2) Synergistic interplay between TRPM2 and NOX2 increases cytoplasmic [Ca^2+^]. (3) Elevated [Ca^2+^] causes lysosomal dysfunction and damage (LMP (Lysosomal Membrane Permeabilisation)). (4) Zn^2+^ from dysfunctional lysosomes is translocated to mitochondria. (5) Increased mitochondrial Zn^2+^ causes mitochondrial damage by inhibiting Complex III, raising mtROS (mitochondrial ROS), increasing fragmentation, and reducing ΔΨmt (mitochondrial membrane potential). (6) mtROS diffuses into the cytoplasm to cause double strand DNA breaks and induce PARP-(Poly (ADP-ribose) polymerase 1) activation. PARP-1 generates ADPR (ADP-ribose) and provides positive feedback to TRPM2, perpetuating the cycle. The cycle involves five key molecular players (TRPM2, Ca^2+^, Zn^2+^, ROS, ADPR) and communication between four compartments (plasma membrane, lysosomes, mitochondria and the nucleus).

Evidence for the unified mechanism depicted schematically in Figure 3 emerges from diverse cellular models of chronic stress: high glucose (diabetic stress mimic) on endothelial cells [112]; free fatty acids (metabolic stress mimic) on pancreatic β-cells [113]; Parkinsonian toxin (1-methyl-4-phenylpyridinium, MPP^+^) on neuroblastoma cells [112]; and oxidative stress (H_2_O_2_) on HEK-293 cells conditionally expressing TRPM2 channels [88,112]. These models respectively represent hypertension, type-2 diabetes, Parkinson’s disease and a generic oxidative stress. Although the number of disease models thus far studied is by no means exhaustive, it represents major groups of NCDs, supporting the proposed mechanism.

This proposed mechanism posits that various extracellular stressors converge on the co-activation of the TRPM2 channel and NOX2 enzyme at the plasma membrane. This initial co-activation establishes a positive feedback loop at the plasma membrane itself: NOX2 generates ROS contributing to TRPM2 activation, while the resulting TRPM2-mediated Ca^2+^ influx provides the necessary Ca^2+^ signal to sustain NOX2 activity. This synergy amplifies the cytoplasmic Ca^2+^ signals. The rise in cytoplasmic Ca^2+^ primarily targets lysosomes, causing de-acidification, dysfunction, and potentially LMP or rupture. This lysosomal damage releases sequestered contents, most critically, labile Zn^2+^, into other parts of the cell, especially to the mitochondria. Within mitochondria, elevated Zn^2+^ directly impairs the ETC, enhancing electron leakage predominantly from Complex III, thereby increasing mtROS (O_2_^•−^) production. This burst of mtROS diffuses into the cytoplasm, activates PARP- in the nucleus, generating ADPR, which activates plasma membrane TRPM2 channels, closing the loop and restarting the ROS amplification cycle. This vicious cycle is presumably repeated multiple times before the cell dies in degenerative diseases.

**Figure 4:**
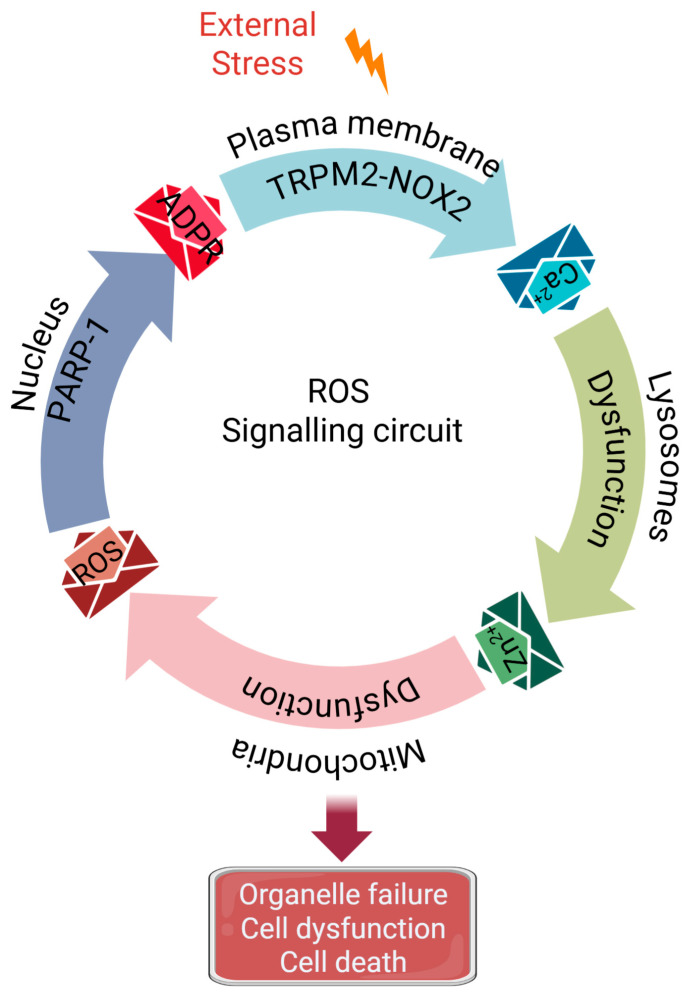
**A Simplified Overview of the ‘Circular Domino Effect’**. A simplified representation of the cytotoxic inter-organelle signalling circuit initiated by stress. Plasma membrane sensing (TRPM2/NOX2) generates Ca^2+^ signals → Ca^2+^ causes lysosomal dysfunction/damage (LMP (Lysosomal Membrane Permeabilisation)) and Zn^2+^ release → Zn^2+^ targets mitochondria (Complex III), causing dysfunction and mtROS production → mtROS (mitochondrial ROS) feeds back to the plasma membrane (TRPM2), by generating ADPR (ADP-ribose) signals from the nucleus (PARP-1, Poly (ADP-ribose) polymerase 1), amplifying the signal and perpetuating organelle damage and eventual cell dysfunction/death. The unique feature of the signalling cascade is its cyclical nature, which presents multiple therapeutic targets: interrupting any node can theoretically mitigate multiple organelle damage, linked to decline in healthspan-to-lifespan ratio as we age.

This mechanism integrates signalling molecules (TRPM2, NOX2, Ca^2+^, Zn^2+^, ADPR, ROS itself) and organelles (plasma membrane, lysosomes, mitochondria and nucleus) implicated in RIRP into a coherent pathway. It proposes a “circular domino effect” (Figure 4) where an initial stressor triggers a cascade of cytotoxic Ca^2+^, Zn^2+^, ROS and ADPR signals relayed between the plasma membrane, lysosomes, mitochondria and the nucleus. During this relay, progressive damage of lysosomes, mitochondria and nuclei occurs, leading to cell dysfunction and or demise. This cycle provides a plausible mechanistic explanation for ROS amplification and organelle impairment, the hallmarks of most NCDs [134]. The subsequent sections dissect the key steps of this putative cycle in more detail, as this aspect has not been previously reviewed.

#### 3.1.1. Initiating the Cycle: Synergistic Activation of TRPM2 and NOX2 at the Plasma Membrane

The cycle begins at the plasma membrane. External stress signals are initially sensed and transduced at the plasma membrane via TRPM2 and NOX2 co-activation. Studies with cellular models of Parkinson’s disease and generic oxidative stress revealed a striking reciprocal dependence between these proteins under stress. NOX2 activation is dependent on TRPM2-mediated Ca^2+^ entry, and conversely, sustained TRPM2 activity (measured by Ca^2+^ influx) required NOX2-generated ROS [88]. Pharmacological inhibition or siRNA knockdown of either TRPM2 or NOX2 abrogated the stress-induced rise in intracellular Ca^2+^ and prevented subsequent ROS amplification and downstream cytotoxicity (lysosomal/mitochondrial damage, cell death) [88]. This suggests that TRPM2-NOX2 interplay establishes a crucial positive feedback loop at the plasma membrane, acting as a highly sensitive trigger that exacerbates Ca^2+^ influx in response to oxidative insults.

However, the precise molecular mechanism of their cooperation and activation sequence remains unclear. Plausible scenarios include: (i) initial stress causes basal TRPM2 activation (via other ROS or direct sensing), generating the initial Ca^2+^ for NOX2 assembly/activation; or (ii) initial stress primarily activates NOX2, whose ROS products then trigger robust TRPM2 opening. Further research is needed to address these possibilities.

Two key observations are noteworthy. First, the apparent channel specificity: despite other ROS-sensitive plasma membrane Ca^2+^ transport mechanisms (other TRPs, SOCE) [98,99,135], TRPM2 downregulation alone abolished the stress-induced cytotoxic cascade in studied models. Second, selectivity for pathogenic ROS levels: inhibiting the TRPM2-NOX2 axis prevented stress-induced ROS amplification but did not appear to affect basal physiological ROS [88]. This specificity, conferred by the Zn^2+^-dependent inhibition of complex III, is therapeutically significant, potentially overcoming the limitation of general antioxidants—their inability to discriminate between beneficial and detrimental ROS [28,29]. Targeting the TRPM2-NOX2 duo (or their interaction) could offer a safer strategy. Elucidating the molecular basis of TRPM2-NOX2 coupling mechanism should be a key goal for future research.

#### 3.1.2. Calcium Overload Targets Lysosomes

It is well accepted that rise in cytoplasmic Ca^2+^ to supraphysiological levels impacts mitochondria to affect its structure and function, leading to abnormal mitochondrial fragmentation, bioenergetic failure and ROS production [38,44,112,113,129]. Recent findings suggest that not all mitochondrial effects of Ca^2+^ are direct. During stress, Ca^2+^ signals mitochondrial damage via lysosomes. In stress models thus far examined, the Ca^2+^ rise consistently caused lysosomal dysfunction (proton gradient dissipation/de-acidification) and physical damage/permeabilization, leading to secondary effects on mitochondria [88,112].

The precise molecular mechanisms by which elevated cytoplasmic Ca^2+^ leads to lysosomal de-acidification, dysfunction, and damage remain to be fully elucidated. Possibilities include effects on the V-ATPase pump, altered lysosomal membrane lipid composition/stability, or activation of Ca^2+^-dependent enzymes compromising integrity. Nevertheless, identifying a specific upstream pathway (stress → TRPM2/NOX2 → Ca^2+^ overload → lysosomal damage) is a fundamental step in unravelling the mechanistic basis for the lysosomal dysfunction and damage widely observed in chronic stress and associated diseases.

#### 3.1.3. From Damaged Lysosomes to Mitochondria: The Journey of Zinc

Lysosomes store a number of factors (e.g., enzymes, metal ions) required for their function, but if unleashed, they can have damaging effects, resulting in cell dysfunction and even cell death [36,37,45,46,52,56,59,60,67,130]. Among the factors that can contribute to abnormal downstream signalling are Ca^2+^ and Zn^2+^. Cells possess robust mechanisms for handling excess cytoplasmic Ca^2+^ (ER sequestration via the Sarcoendoplasmic Reticulum Calcium ATPase pump, mitochondrial uptake via mitochondrial uniporter, plasma membrane efflux via plasma membrane Ca^2+^-ATPase/sodium-calcium exchanger), to restoring basal levels quickly after transient increases to prevent Ca^2+^-induced damage [98,99,136].

However, compared to Ca^2+^ homeostasis, how cells handle sudden release of labile cytoplasmic Zn^2+^ is less understood. While the cytoplasm contains significant amounts of high-affinity Zn^2+^-binding metallothioneins (primary buffer), studies found mitochondria nonetheless accumulated significant Zn^2+^ following the Ca^2+^-dependent lysosomal damage [88,112,113]. This mitochondrial Zn^2+^ accumulation was observed consistently across different models of cellular stress representing different disease conditions. The precise mechanism by which released Zn^2+^ bypasses/overwhelms cytoplasmic buffering and selectively translocates into mitochondria remains to be determined.

Genetic evidence from studies of mutations associated with Parkinson’s disease underpins the pathogenic importance of mitochondrial translocation of lysosomal Zn^2+^ [57,69]. Notably, studies of *PARK9* mutations demonstrated redistribution of lysosomal Zn^2+^ to mitochondria. The mutation impairs the function of the *PARK9*-encoded ATP13A2 (a lysosomal Zn^2+^ importer), resulting in mitochondrial Zn^2+^ accumulation, subsequent mitochondrial fragmentation, dysfunction and ROS production. These findings underscore the importance of Zn^2+^ accumulation in mitochondrial damage. As with the genetic condition, the source of mitochondria-damaging Zn^2+^ during chronic stress is lysosomes, and the stimulus for this is extracellular Ca^2+^ entry [88,112,113]. Thus, redistribution of lysosomal Zn^2+^ to mitochondria may be an important step in pathogenic mechanisms associated with chronic stress.

#### 3.1.4. Zinc Disrupts Mitochondrial Function and Bolsters ROS Production

As demonstrated with *PARK9* mutations, the Ca^2+^-driven mobilisation of lysosomal Zn^2+^ to mitochondria led to the loss of ΔΨmt, increased ROS production, and mitochondrial fragmentation [88,112,113]. Notably, chelation of Zn^2+^ with TPEN (N,N,N′,N′-Tetrakis(2-pyridylmethyl)ethylenediamine) mitigated the mitochondrial events triggered by the Ca^2+^-induced lysosomal damage [88,112,113]. These findings led to the conclusion that the detrimental effects of Ca^2+^ on mitochondria are not direct, but mediated by Zn^2+^, positioning Zn^2+^ downstream of Ca^2+^.

These findings might warrant a review of the long-accepted mechanism for how Ca^2+^ causes mitochondrial damage, by alluding to the fact that the effect of Ca^2+^ on mitochondria is mediated by Zn^2+^ during chronic stress. Previous studies might have missed this due to the reliance on BAPTA (1,2-Bis(2-Aminophenoxy)ethane-N,N,N′,N′-tetra acetic acid) to probe Ca^2+^-driven events, as BAPTA binds Zn^2+^ with much greater affinity (≥10-fold) than Ca^2+^ [115,122].

While Ca^2+^ can stimulate ROS generation from multiple mitochondrial sites, elevated mitochondrial matrix Zn^2+^ can directly stimulate electron leak from Complexes I and III, increasing ROS production [137,138]. Using compounds capable of quenching electron leak from Complex I (S1QEL) and Complex III (S3QEL), recent studies demonstrated S3QEL is far more effective than S1QEL in suppressing ROS production in neuroblastoma cells subjected to treatments that raised mitochondrial Zn^2+^ [88]. The stronger S3QEL effect seems consistent with Complex III having a high-affinity (K_i_ ~0.1 µM) [137,138] Zn^2+^ binding site near the Qo electron transfer site [89]. The weaker S1QEL effect aligns with reports that Complex I lacks a Zn^2+^ binding site in its catalytic core and has relatively low affinity (IC_50_ ~10–50 µM) [137,138]. In addition, basal mitochondrial Zn^2+^ is extremely low (picomolar) [139], so any rise would arguably inhibit Complex III before affecting Complex I.

Compelling evidence for Complex III’s unique pathogenic role comes from in vivo knockout mice studies. Genetic deletion of functionally important subunits in neurons revealed Complex III, but not other complexes, contributes to ROS damage, neuronal cell death, and motor deficits [85,87]. More recently, the group demonstrated altered redox status, early β-cell dysfunction, and hyperglycaemia in Complex III-depleted mice [86]. Furthermore, a recent genome-wide study linked mutations in mitochondrial UQCRC1 (a core Complex III component) to Parkinson’s disease [84]. Knock-in of these mutations in Drosophila and mouse models showed age-dependent locomotor deficits and dopaminergic neuronal death [84]. In neuroblastoma cells, these mutations affected mitochondrial function, increasing ROS [84]. Determining if Complex III plays a similar role in other pathologies, alongside the role of lysosomal Zn^2+^ redistribution to mitochondria, is important.

#### 3.1.5. Closing the Loop: Mitochondrial ROS Stimulates ADPR Production to Perpetuate the Cycle

The final crucial step is feedback from damaged mitochondria back to the plasma membrane to sustain initial TRPM2-NOX2 activation. Increased mtROS (from mitochondrial Zn^2+^ accumulation and Complex III inhibition) can diffuse out and reach the cytoplasm, further stimulating TRPM2 channel activity by generating the TRPM2 agonist ADPR. Although ADPR can be generated both in the mitochondria and nucleus, in chronic stress, much of it is generated in the nucleus through PARP-1 activation due to increased nuclear DNA damage [140]. This renewed TRPM2 activation ensures continued Ca^2+^ influx, sustaining NOX2 activity and promoting lysosomal damage, thus closing the positive feedback loop. Scavenging the mtROS with mito-TEMPO abolishes TRPM2-mediated cell death, as effectively as S3QEL and PJ34 [88], supporting the feedback effect of mtROS on TRPM2.

Thus, in addition to the feedback loop between TRPM2 and NOX2 at the plasma membrane, there is a second feedback activation loop from mtROS on the TRPM2 channel, involving ADPR generation in the nucleus from PARP-1 activation. This indicates that the communication between mitochondria and the plasma membrane occurs through the nucleus. The cycle entails sustained generation of distinct signals (at each organelle) that enable signal transmission from the plasma membrane through lysosomes (Ca^2+^), mitochondria (Zn^2+^), nucleus (mtROS) and back to the plasma membrane (ADPR). The built-in feedback loops enable amplification of signals at different nodes within the cycle, leading to progressive damage of lysosomes, mitochondria and nuclei, and eventually to cell dysfunction and demise.

### 3.2. The Impact of Activating the Signalling Cycle

The pathogenic effect of activating this signalling cycle depends on the cell type and its physiological role. Effects can range from accelerated ageing to chronic conditions including diabetes and cardiovascular and neurodegenerative diseases. Few studies have systematically investigated interrupting the cycle at various points—inhibiting TRPM2 or NOX2, chelating Ca^2+^ or Zn^2+^, preventing Complex III electron escape, scavenging mtROS, or inhibiting PARP-1—on organelle damage and pathophysiological consequences (e.g., programmed cell death) across different disease models to determine the pathway’s broad significance [88,113]. Programmed cell death (apoptosis or parthanatos) results from the release of cytochrome *C* and AIF (apoptosis-inducing factor) from the damaged mitochondria, and cathepsins from damaged lysosomes.

For example, studies on pancreatic β-cells (diabetes model) and neuronal cells (PD model) concurrently examined roles for NOX2, TRPM2, Ca^2+^, Zn^2+^, and Complex III (in PD model only) in lysosomal/mitochondrial damage and ROS amplification, demonstrating the circuit’s role in cell death [88,113]. In human endothelial cells, diabetic stress led to TRPM2-mediated Ca^2+^ influx, lysosomal impairment, Zn^2+^ translocation to mitochondria, Drp1-dependent mitochondrial damage, and ROS production [112]. Other studies link excess mitochondrial fragmentation in this model to reduced nitric oxide generation, relevant to hypertension regulation [43].

Thus, akin to a “circular domino effect,” the signalling circuit involves sequential, reciprocal lysosomal, mitochondrial and nuclear damage, initiated by plasma membrane Ca^2+^ signals (TRPM2-NOX2 co-activation) and propagated by Zn^2+^ (lysosome-generated), ROS (mitochondria-generated) and ADPR (nucleus-generated) signals culminating in pathogenic responses (Figure 3 and Figure 4). The cycle provides a plausible mechanistic explanation for how oxidative stress becomes amplified and self-sustaining, leading to progressive organelle damage and pathological outcomes in chronic disease settings.

### 3.3. Evidence Supporting the Unified Mechanism Across Disease Models

Support for this unified mechanism comes from studies using diverse cellular and in vivo models relevant to human OS-associated diseases. For instance, exposing human endothelial cells to high glucose (diabetic stress mimic) induced lysosomal damage, mitochondrial fragmentation/dysfunction, and excessive ROS—linked phenotypically to reduced nitric oxide bioavailability and endothelial dysfunction [43,112]. Crucially, these phenotypic effects were attenuated/prevented by pharmacological inhibitors of TRPM2, genetic suppression of TRPM2 (siRNA), PARP-1 inhibition with PJ34, chelation of intracellular Ca^2+^ (BAPTA-AM), chelation of intracellular Zn^2+^ (TPEN) and sequestration of ROS.

Similarly, exposing rodent and human pancreatic β-cells to excess free fatty acids (metabolic stress mimic) led to a comparable cascade: mitochondrial fragmentation/dysfunction, ROS overproduction, and β-cell apoptosis [113]. These outcomes were rescued by RNAi-mediated knockdown or pharmacological inhibition of TRPM2, PARP inhibition, and by Ca^2+^/Zn^2+^ chelators and ROS quenchers. In both models, evidence indicated Ca^2+^ overload caused initial lysosomal damage, while subsequent mitochondrial damage depended on the downstream increase in mitochondrial Zn^2+^. Furthermore, NOX2 inhibition abolished free fatty acid-induced ROS overproduction in β-cells, implicating NOX2 in initiation of the cycle at the plasma membrane. Supporting in vivo relevance, rodent models of high-fat diet-induced obesity/diabetes showed that genetic knockout of *Trpm2* prevented hyperglycaemia, preserved mitochondrial function, and attenuated weight gain [141].

More recently, a similar mechanism was confirmed in a cellular model of Parkinson’s disease. Treating neuroblastoma cells with MPP^+^ co-stimulated TRPM2 and NOX2, leading to increased intracellular Ca^2+^. This Ca^2+^ rise triggered lysosomal damage, followed by Zn^2+^-dependent mitochondrial damage (fragmentation, dysfunction), ROS amplification, and cell death. In addition, PARP-1 inhibition mitigated the phenotypic effects of the toxin [88].

Remarkably, the core phenotypic changes across these diverse stress models (high glucose, fatty acids, MPP^+^) could be largely recapitulated in a simpler, recombinant HEK-293 cell system conditionally expressing TRPM2 using the generic oxidant H_2_O_2_ as the stressor, suggesting a conserved fundamental pathway. A notable feature of this proposed destructive cascade is its self-perpetuating nature: organelles generate signals that cause their own damage.

Although the number of disease models studied to arrive at the signalling circuit are somewhat limited, there are numerous reports in the literature supporting the individual steps within the cycle in a diverse range of in vitro and in vivo disease models. An examination of these studies implicating two or more of the signalling molecules (TRPM2, NOX2, PARP1, Zn^2+^ and mitochondrial ROS, -summarised in Table 1) lends circumstantial support to the unified signalling circuit.

Activation and/or upregulation of TRPM2, NOX2, and PARP-1 have been linked to many cancers [114,142,143,144]. While a detailed discussion of this topic is beyond the scope of this review, key findings are summarized in Table 1. TRPM2 activation promotes cell proliferation and preserves cell viability by activating transcription factors and signalling pathways [114]. The role of Zn^2+^, however, is less clear, but TRPM2-mediated lysosomal Zn^2+^ release promotes mitochondrial fission, which is required for inheritance and partitioning of mitochondria during cell division [116,145,146]. It is therefore reasonable to assume that Zn^2+^ plays a role in cell proliferation. Inhibition of mitochondrial fission has been shown to prevent cell cycle progression in cancer [147]. In addition to promoting cell proliferation, TRPM2 activation facilitates Zn^2+^-dependent remodelling of the actin cytoskeleton and focal adhesions, enhancing cell migration [115]. Furthermore, studies have shown activation of PARP-1 and NOX2 promotes migration of several cancer cell types [148,149]. However, it should be noted that the ROS-dependent signalling mechanisms are highly complex and vary significantly depending on the cancer type.

Although comprehensive, systematic studies linking all the events of the cycle to individual NCDs and cell types are required, the comparison of existing reports (Table 1) lends circumstantial support to the idea that this signalling axis is likely conserved among many OS-linked diseases.

However, significant further research is required. Key areas include: elucidating precise TRPM2-NOX2 coupling mechanisms; understanding how Ca^2+^ damages lysosomes; identifying pathways for lysosomal Zn^2+^ release and mitochondrial Zn^2+^ uptake under stress; confirming Complex III’s primary role as the Zn^2+^-sensitive mtROS source; and validating the cycle’s operation and therapeutic tractability in more complex in vivo models and human pathophysiology. Addressing these questions is crucial for translating these exciting mechanistic insights into effective clinical therapies for combating OS-associated diseases.

## 4. Therapeutic Opportunities and Future Directions

While cyclical signalling mechanisms are crucial for fundamental rhythmic and cell division processes [150,151], they represent a smaller subset of the overall signalling landscape in biology. Linear signalling cascades, with their inherent flexibility and capacity for diverse responses, constitute the vast majority of the intricate communication networks within and between cells. These linear pathways can sometimes incorporate feedback loops that introduce oscillatory behaviour in downstream targets, but the core signal transduction mechanism is typically a linear progression of molecular events.

Unlike common linear signalling pathways, the pathway described here (Figure 4) seems to operate in cyclical fashion, akin to metabolic cycles. A key advantage of such a cycle over linear pathways is that it offers a rich landscape for therapeutic intervention. One could intervene at any one of the available nodes and the entire cycle stops. Indeed, as explained above, inhibiting any of the molecular players-TRPM2, NOX2, mitochondrial complex III, or PARP-1-or scavenging the signals they generate (Ca^2+^, Zn^2+^ and mtROS), mitigates lysosomal, mitochondrial and nuclear damage, and cell demise-the hallmarks of most NCDs. The chemicals used to interrupt the cycle, however, are not suitable for safe therapeutic use, but provide the proof-of-principle that many of the nodes in the cycle are potential drug targets.

Any therapeutic developed based on this cycle is unlikely to exhibit significant adverse effects because it would selectively interrupt pathological ROS production, sparing essential signalling ROS production (unlike general antioxidants). Targeting TRPM2 directly holds promise. Similarly, developing highly selective NOX2 inhibitors could break the cycle at initiation, though isoform selectivity remains challenging. Preventing lysosomal damage or stabilizing membranes could stop toxic Zn^2+^ release. Alternatively, specific intracellular Zn^2+^ chelators accessing relevant compartments, or strategies preventing mitochondrial Zn^2+^ uptake, could protect mitochondria. Finally, targeting mtROS production itself, specifically reducing Complex III electron leak, could prevent feedback amplification. PARP-1 inhibitors are used clinically for cancer treatment [142,143,152] and are being currently targeted for therapeutic treatment of NCDs [153].

The potential utility of targeting this cycle is underscored by its apparent involvement across multiple disease models (diabetes complications, neurodegeneration, potentially others involving chronic oxidative stress). This suggests interventions aimed at this core TRPM2-Ca^2+^-Lysosome-Zn^2+^-Mitochondria-ROS axis could represent broad-spectrum therapeutics for the underlying OS common to numerous NCDs. By preventing the vicious cycle, such therapies might treat specific diseases and potentially slow aspects of aging, enhancing the healthspan-to-lifespan ratio.

**Table 1:** Compilation of Literature Illustrating the Shared Roles of Signalling Proteins and Molecules Supporting the Unified Mechanism.

Disease	TRPM2 Involvement	NOX2 Involvement	Zn^2+^ Involvement	Mitochondrial ROS Involvement	PARP Involvement
**Alzheimer’s Disease (AD)**	TRPM2 inhibition (2-APB or ACA): ↓Aβ42-induced neuronal death in mouse hippocampus [154]. TRPM2 KO in mice: ↓Aβ-induced neurotoxicity ↓Ca^2+^ influx ↓TNF-α release [154]. TRPM2 KO (APP/PS1 mice): Improved spatial memory, ↓Microglial activation in hippocampus [155].	NOX2 KO in mice: improved spatial memory [156]. Postmortem analyses: ↑NOX2 activity and expression in frontal and temporal cortices in patients with mild cognitive impairment [157].	Zn^2+^ chelation (Clioquinol): Potential in reducing plaque load in AD models [158]. ZnT3-deficient mice: ↓Aβ oligomer accumulation [158].	Scavenging mito-ROS with mitochondria targeted ROS scavengers in 3xTg-AD mice: ↓Oxidative stress ↓Aβ oligomer accumulation ↓Cell death ↓Cognitive impairment [159].	PARP inhibition (pharmacological and genetic): ↓neuronal loss through parthanatos, neuroinflammation, cognitive impairment [153].
**Parkinson’s Disease (PD)**	TRPM2 inhibition (2-APB, PJ34) and siRNA silencing in a cellular model: ↓MPP^+^- induced mtROS production and cell death [88,160]. Post-mortem brains of PD patients: ↑TRPM2 protein levels in SNpc [160].	NOX2 inhibition (chemical) in a cellular model: ↓MPP^+^-induced Ca^2+^ rise ↓mtROS production ↓Cell death [88]. NOX2 inhibition (apocynin) in paraquat and 6-OHDA administered mice: ↓Cognitive deficits ↓Oxidative stress ↓Neuroinflammation [161,162]. NOX2 KO in 6-OHDA administered mice: ↓Dopaminergic neuron loss [162]. Post-mortem brains of PD patients: ↑gp91^phox^ expression in midbrain [163].	Zn^2+^ chelation (TPEN): ↓ROS levels ↓MPP^+^-induced cytotoxicity [88]. Zn^2+^ chelation (Clioquinol) in Lewy Bodies-injected mice: ↓α-synuclein-associated degeneration [164]. Post-mortem brains of PD patients: ↑Zn^2+^ levels observed in SNpc [165]. Genetic mutations in *PARK9*: ↑Mitochondrial Zn^2+^ in dopaminergic neurons ↑Mitochondrial damage [57,69].	Scavenging mito-ROS with mitochondria- targeted ROS scavengers: ↓MPP^+^-induced cell death [88]. MitoQ in preclinical models: Neuroprotective [166].	PARP-1 chemical inhibition or KO in mice: ↓α-synuclein-induced toxicity and neuronal cell death [167]. Post-mortem PD patient brains and CSF: ↑PAR levels [167].
**Cardiac ischemia**	TRPM2 inhibition (chemical) or KO in mice subjected to IR injury: ↓Infarct size ↓Inflammation ↑Cardiac outcome [168].	NOX2 KO in mice subjected to IR injury: ↓Infarct size [169].	Zn^2+^ chelation (TPEN) in rat hearts during IR injury: ↓Infarct area [170].	Scavenging mito-ROS with MitoQ in rats subjected to IR injury: ↓Mitochondrial damage ↓Cell death ↑Cardiac function [171].	PARP1 inhibition (chemical) in mice subjected to IR injury: ↓Infarct size ↓Inflammation ↑Cardiac function [172].
**Stroke/Cerebral Ischemia**	TRPM2 KO or inhibition (chemical) in male mice subjected to IR injury: ↓Neuronal cell death ↓Infarct size ↓Memory loss [173,174].	NOX2 KO in mice subjected to IR injury: Delay infarct progression, but no protection from brain injury [175].	Zn^2+^ chelation (TPEN): Protects mice from ischaemic brain damage [118].	Mitochondrial ROS in IR injury mouse model: ↑Mitochondrial ROS in hippocampus in mice. MitoQ: ↓Hippocampal damage [176].	PARP1 gene inactivation: Protection against ischemic insults [177].
**Various Cancers**	TRPM2 Inhibition (chemical/genetic: SiRNA/KO): Breast cancer cells ↓Proliferation ↑DNA damage [178]. Neuroblastoma cells ↓Viability ↑ROS ↑DNA damage (sensitised to doxorubicin) [179]. Leukaemia ↓Proliferation ↑Chemo sensitivity [180]. Ovarian Cancer ↓Cell viability ↓Proliferation ↑Apoptosis [181]. PC3 and HeLa ↓Cell migration [115].	NOX2 inhibition: Leukaemia cells ↑Cell death [182]. NOX2-KO and inhibition in mice: ↓Lung metastases [183].	Zn^2+^ depletion: Breast cancer cells ZIP10 KO or zinc depletion: ↓Cell migration [184]. Zn^2+^ chelation (TPEN) in PC3 and HeLa: ↓Cell migration [115].	Scavenging mito-ROS in mice: Mice lung carcinoma cells ↓Metastasis [185]. Scavenging mito-ROS in mouse melanoma cells ↓Cell growth ↓viability ↑Apoptosis [186].	↑PARP1 expression in breast, ovarian, and lung cancers. [143]. PARP1 inhibition in cervical cancer cell lines: ↓Proliferation ↑Cell death ↓Metastasis [187]. PARP1 inhibition in liver cancer cells: ↓Proliferation ↓Cell migration [188]. PARP inhibition in PC3 and HeLa: ↓Cell migration [115].
**Atherosclerosis (AS)**	TRPM2 KO in Apoe-/- mice: ↓Progression of AS [189]. TRPM2 inhibition, KO and KD: ↓Mitochondrial damage in EC [112].	NOX2 KO in Apoe/-e mice: ↓Plaque formation due to absence of NOX2 in macrophages and vessel wall cells [190].	Data not available	MitoQ treatment HFD Apoe-/- mice: ↓Macrophages in plaques ↓Cell proliferation macrophages in plaques [191].	PARP1 inhibition or KO Apoe/-e mice: ↓Plaque formation ↓Progression of atherosclerosis [192].
**Type 2 Diabetes**	TRPM2 KO in HFD mice: ↑Insulin Sensitivity ↑Resistance to Diet-Induced Obesity ↑Glucose Metabolism ↓Inflammation [141]. Pancreatic β-cells (FFA treated): ↑NOX-dependent ROS ↑Mitochondrial damage ↑Cell death [113].	NOX2 KO: ↑Insulin Sensitivity ↑Resistance to Diet-Induced Obesity [193]. NOX2 KD using SiRNA in pancreatic β-cell line exposed to high glucose and FFA: ↑ β-cell function ↑ β-cell survival [194,195]	Zn^2+^ chelation (TPEN): ↓FFA -induced β-cell death [113]. Loss of function mutations in *hZnT8 in* humans: ↑Risk of T2D ↑β-cell survival [196] Overexpression of LoF *hZnT8* mutant in HFD-mice: ↑Glucose tolerance [197].	Excess nutrition: ↑mtROS production ↑Insulin resistance ↑β-cell dysfunction [198].	PARP-1 KO: ↓β-cell dysfunction ↓Insulin resistance ↓Vascular damage [199]. PARP-1 inhibition (PJ34) in pancreatic β-cells: ↓Cell death [113].
**Type 1 diabetes**	TRPM2 KO in mice (STZ model): ↓β-cell death ↓Hyperglycaemia [200].	NOX2 KO: ↓Glucose-induced superoxide in islets ↑Glucose-induced insulin secretion ↓β-cell apoptosis [201].	Zn^2+^ chelation in STZ mouse model: ↓β-cell death ↓Hyperglycaemia [202] Pancreatic β-cells: Zn^2+^ chelation prevents oxidant- induced β-cell death [200].	Mitochondrial ROS: ↑β-cell damage from cytokines and immune cells [23].	PARP-1 KO in STZ mouse model: ↓β-cell death ↓Hyperglycaemia [203].

Abbreviations: Aβ (Amyloid-beta), ACA (Arachidonyltrifluoromethyl ketone), 2-APB (2-aminoethoxydiphenyl borate), CSF (Cerebrospinal Fluid), EC (Endothelial Cells), FFA (Free Fatty Acids), HFD (High fat diet), IR (Ischemia-Reperfusion), KO (Knockout), KD (Knockdown), LoF (Loss of function), MPP+ (1-methyl-4-phenylpyridinium), mtROS (mitochondrial ROS), 6-OHDA (6-Hydroxydopamine), PAR (Poly(ADP-ribose), PARP (Poly(ADP-ribose) polymerase, PJ34 (N-[2-(4-Pyridinyl)-1H-indol-3-yl]methanesulfonamide), TPEN (N,N,N′,N′-Tetrakis(2-pyridylmethyl)ethylenediamine), SNpc (Substantia Nigra pars compacta), STZ (Streptozotocin), PC3 (Prostate Cancer Cell line). ↑ (increase/stimulation/worsening of pathology); ↓ (decrease/inhibition/amelioration of pathology).

## 5. Conclusions

In conclusion, this review has synthesized evidence from diverse fields to propose a unified, self-perpetuating vicious cycle as a central driver of pathological oxidative stress in numerous non-communicable diseases. This ‘circular domino effect’ connects plasma membrane stress-sensing (TRPM2/NOX2) to a cascade of inter-organelle dysfunction, orchestrated by ionic signals (Ca^2+^, Zn^2+^) and redox messengers (ROS, ADPR). By integrating the plasma membrane, lysosomes, mitochondria, and nucleus into a single coherent pathway, this model provides a plausible mechanistic explanation for how an initial stressor can trigger sustained ROS amplification, progressive organelle failure, and ultimately, cellular demise—the hallmarks of many NCDs and the aging process itself. The cyclical nature of this pathway presents a paradigm shift for therapeutic intervention. Unlike linear pathways, targeting any single node—be it TRPM2, NOX2, Complex III, or PARP-1—has the potential to break the entire amplification loop. Crucially, this targeted approach offers the promise of selectively disabling pathological ROS production while preserving essential physiological redox signalling, thereby overcoming the limitations of non-specific antioxidant strategies. Further elucidation and validation of this TRPM2-NOX2-mediated axis will be paramount. It not only opens novel avenues for developing broad-spectrum therapeutics against a host of debilitating diseases but also holds the potential to slow the functional decline associated with aging, ultimately aiming to enhance the human healthspan-to-lifespan ratio.
